# Characterization of four virulent Klebsiella pneumoniae bacteriophages, and evaluation of their potential use in complex phage preparation

**DOI:** 10.1186/s12985-020-01485-w

**Published:** 2021-01-06

**Authors:** Fedor Zurabov, Evgeniy Zhilenkov

**Affiliations:** 1 Research and Production Center “MicroMir”, LLC, Moscow, Russia; 2 Department of Virology, Lomonosov Moscow State University, Moscow, Russia

**Keywords:** *Klebsiella pneumoniae*, Bacteriophages, Phage cocktails, ESKAPE, Phage therapy, Depolymerases, Biofilms

## Abstract

**Background:**

Nowadays, hundreds of thousands of deaths per year are caused by antibiotic resistant nosocomial infections and the prognosis for future years is much worse, as evidenced by modern research. Bacteria of the *Klebsiella* genus are one of the main pathogens that cause nosocomial infections. Among the many antimicrobials offered to replace or supplement traditional antibiotics, bacteriophages are promising candidates.

**Methods:**

This article presents microbiological, physicochemical and genomic characterization of 4 virulent bacteriophages belonging to *Siphoviridae*, *Myoviridae* and *Podoviridae* families. Phages were studied by electron microscopy; their host range, lytic activity, adsorption rate, burst size, latent period, frequency of phage-resistant forms generation, lysis dynamics and sensitivity of phage particles to temperature and pH were identified; genomes of all 4 bacteriophages were studied by restriction digestion and complete genome sequence.

**Results:**

Studied phages showed wide host range and high stability at different temperature and pH values. In contrast with single phages, a cocktail of bacteriophages lysed all studied bacterial strains, moreover, no cases of the emergence of phage-resistant bacterial colonies were detected. Genomic data proved that isolated viruses do not carry antibiotic resistance, virulence or lysogenic genes. Three out of four bacteriophages encode polysaccharide depolymerases, which are involved in the degradation of biofilms and capsules.

**Conclusions:**

The bacteriophages studied in this work are promising for further in vivo studies and might be used in phage therapy as part of a complex therapeutic and prophylactic phage preparation. The conducted studies showed that the complex preparation is more effective than individual phages. The use of the complex phage cocktail allows to extend the lytic spectrum, and significantly reduces the possibility of phage-resistant forms generation.

## Background

Nowadays, increased incidence of multidrug resistance in bacterial pathogens is one of the most important medical and social problems. Uncontrolled usage of antibiotics led to the emergence of multidrug resistant bacterial strains and resulted in limited efficacy of treatment with current antibiotics, and a high probability of patient colonization by resistant strains. Absence of drugs against bacterial infections or their inefficiency is a serious problem of the twenty-first century [1]. In 2009, more than 100 thousand people died worldwide from bacterial infections in the absence of effective antibiotics. The situation is deteriorating, and as of 2018, the number of annual deaths worldwide has reached 700 thousand [2]. The predictions are worrying: it is estimated that 10 million people will die from antibiotic-resistant infections in 2050 if nothing changes [3, 4].

The situation is complicated by the fact that the development of new antibacterial agents is absent due to the too long and complicated process of their creation and licensing, which is unacceptable in the context of the global crisis. Pharmaceutical companies are abandoning the development of new antibiotic drugs because of the risk of the onset of antibiotic resistance [5]. Thus, ESKAPE microorganisms (*Enterococcus faecium*, *Staphylococcus aureus*, *Klebsiella pneumoniae*, *Acinetobacter baumannii*, *Pseudomonas aeruginosa* and *Enterobacter*) are characterized by rapid acquisition of resistance to various antimicrobial agents, including multidrug resistance [6].

Antibiotics are not only becoming less effective, but their use can also cause dysbiosis, especially in the intestines or in places of secondary infections. The effects on human health can be catastrophic: excessive reuse of antibiotics has been shown to destroy most of the natural intestinal flora [7].

Thus, it is currently necessary to develop alternative methods to control bacteria that are resistant to antibiotic drugs. Among the many antimicrobial agents offered to replace or supplement traditional antibiotics, bacteriophages are promising candidates, as evidenced by the National Health Institute (NIH, USA), which marked the potential of these biological agents in the fight against antibiotic resistance [8].

The *Klebsiella* genus belongs to the *Enterobacteriaceae* family. *Klebsiella pneumoniae* is a Gram-negative, nonmotile, encapsulated rod-shaped bacterium. It is associated with pneumonia, urinary tract infections and nosocomial infections. Respiratory tract infections are among the most common and severe infections in the world. *Klebsiella pneumoniae* is one of the main pathogens that cause respiratory tract infections in humans. The clinical picture of such diseases is rapidly progressing, which leads to a high mortality rate (up to 50%) [9, 10]. The situation worsens because 80% of nosocomial infections are caused by strains resistant to antibiotics, which complicates their treatment. [11].

Antibiotic resistance, including multidrug-resistance, can develop due to the presence of plasmids carrying the antibiotic resistance genes (R plasmids) [12]. For example, resistance to β-lactam antibiotics can be encoded by plasmid genes and occurs through the synthesis of carbapenem-hydrolyzing β-lactamases, enzymes that hydrolyze β-lactam antibiotics [13]. Strains of *Klebsiella pneumoniae* are often resistant to extended-spectrum beta-lactams (e.g. penicillins and cephalosporins). Still, extended-spectrum beta lactamase (ESBL)-producing strains of *Klebsiella pneumoniae* remain sensitive to the carbapenem antibiotic class (e.g. imipenem and meropenem). However, there is growing incidence of *Klebsiella pneumoniae* infections caused by strains which have become resistant even to carbapenems. This occurred due to the increased use of carbapenem class antibiotics against ESBL-producing strains of *Klebsiella pneumoniae* [14–16]. Bacteriophage therapy can be a method of combating infections caused by antibiotic-resistant strains of *Klebsiella pneumoniae* [17].

*Klebsiella pneumoniae* also produces various extracellular polysaccharides such as lipopolysaccharides (LPS), capsule polysaccharides (CPS), and exopolysaccharides (EPS), which play an important role in protecting bacterial colonies against antibiotics [18, 19] and preventing phages from accessing the receptors [20]. LPS consist of lipid A, core and O-polysaccharide antigen. LPS are located on the bacterial outer leaflet of the outer membrane and provide protection against antimicrobial peptides and the complement system [21, 22]. CPS form the outer layer, consist of linear or branched oligosaccharides and represent a physical barrier, which protects from phagocytosis and masks the receptors for phages [20, 23]. Both CPS and LPS participate in biofilm formation. LPS are necessary for initial adhesion on abiotic surfaces, and CPS are necessary for initial substrate coverage and construction of mature biofilm architecture [24]. The outer cell matrix, which is a component of biofilms of *Klebsiella* bacteria, consists of protein adhesives, nucleic acids and CPS [25]. It has been shown shown that EPS prevent the action of antimicrobial peptides of the host's immune system [26].

Although the capsules can protect bacteria from phage infection by masking host cell receptors, some bacteriophages, in contrast, can use these polysaccharide shells as receptors to initiate infection [20]. Such phages usually have multiple copies of tail fibrils or spikes of different types, each capable of destruction of a particular type of capsule [27]. The sequential disintegration of polymer bonds without dissociation of the virus allows virions to travel through the capsule layer until they reach the secondary receptors [28]. Bacteriophages can also encode exopolysaccharide depolymerases to facilitate biofilm penetration and infection of resident bacteria [29, 30]. Polysaccharide depolymerases play an important role in biofilm degradation, allowing viruses to be more effective [31]. Depolymerases are very useful because of their ability to attack carbohydrates of bacterial membranes [32, 33] and capability of biofilm degradation. They can be presented in two forms: as a component of a phage particle attached to a basal plate, or as a protein released after phage replication. On soft agar, phages with depolymerase activity often produce a halo surrounding the phage plaques. They are the result of depolymerase diffusion as part of new virions or as free enzymes [30].

Bacteriophages have several advantages over antibiotics, including specificity, safety, efficacy against bacteria with multiple antibiotic resistances, and the capability of biofilm degradation. Moreover, phages are eliminated in the absence of a host bacterium, which makes them the only therapeutic agent that self-regulates at the sites of infection [2]. In addition, a study conducted between 2008 and 2010, which included 153 patients, showed that bacteriophages do not cause an undesirable immune response [34–36].

After isolation, each new phage must be described before it is included in the collection. It is necessary to study such parameters as the latent period, phage burst size [37], adsorption dynamics, storage stability, phage morphology, ultrastructure and taxonomy, as well as the level of generation of phage-resistant forms [38]. For therapeutic purposes, it is worth selecting phages with a minimum level of generation of phage-resistant forms of bacteria. To reduce it, it is recommended to use mixtures of bacteriophages (“cocktails”) [39]. Moreover, bacteriophages should be characterized by sequencing the entire genome to exclude the presence of toxin genes, virulence factor genes, as well as genes responsible for lysogenesis. For the safe use of phages, temperate bacterial viruses must be excluded from the preparations, since the incorporation of phage into the cell genome can lead to the conversion and, for example, production of toxins [40].

This manuscript focuses on *Klebsiella pneumoniae* phages, their polysaccharide depolymerases and potential as alternative antimicrobials, as well as on comparison of the effects of individual phages and complex phage preparations. All bacteriophages were isolated from sewage waters in Moscow, Russia. Their morphology was assessed using transmission electron microscopy, thermal and pH stability were evaluated, one-step-growth parameters and host adsorption rate were characterized, and the complete genome was sequenced and analyzed. Furthermore, the host range and phage-resistant forms generation rate were characterized for both single-phage and complex preparations using clinical strains of *Klebsiella pneumoniae* isolated from different hospitals in Moscow, Russia.

## Materials and methods

### Bacteria isolates

Clinical strains of *Klebsiella pneumoniae* were obtained from the "MicroMir" collection. All strains were examined on a MALDI-TOF Microflex mass spectrometer (Bruker, US) and with biochemical tests (MIKROLATEST, Erba Mannheim) with further analysis on Multiskan Ascent spectrophotometer (Thermo Scientific, US) before their adding to collection. Strains were obtained from:

- Clinical isolates from Tula; Serpukhov; Moscow; Pushchino; city ​​of Ufa;
- Kulakov Scientific Center of Obstetrics, Gynecology and Perinatology;
- Moscow City Scientific Research Institute of Emergency Care named after N. V. Sklifosovsky;
- Clinical Hospital No. 83 of FMBA, Moscow;
- City Clinical Hospital No. 67 in Moscow.

Strains are clinical and were obtained from many patients over several years, which allows us to talk about the relevance of the collection and its compliance with the current spectrum of circulating strains. More detailed information about characteristics of the bacterial strains, including MALDI-TOF and MIKROLATEST results, is included in Additional File 1.

To isolate and produce bacteriophages and to conduct experiments, four strains were used: Kl 327, Kl 325, Kl 315 and Kl 263. These strains are used in the laboratory as the most convenient for production and the most sensitive to bacteriophages. A separate strain was used for each bacteriophage: Kl 327 for vB_KpnS_FZ10, Kl 325 for vB_KpnS_FZ41, Kl 315 for vB_KpnP_FZ12 and Kl 263 for vB_KpnM_FZ14.

### Isolation of phages

*Klebsiella* phages vB_KpnS_FZ10, vB_KpnP_FZ12, vB_KpnM_FZ14 and vB_KpnS_FZ41 were isolated in 4 separate enrichments from sewage water collected from a waste-water treatment plant near hospital №5 located in Chekhov district, Moscow region, Russia. The isolation process began with the addition of sodium phosphate buffer (pH 7.0) to the sample to a final concentration of 0.05 M, and NaCl was added to a final concentration of 1 M. The mixture was incubated on an Environmental Shaker-Incubator ES-20/60 (Biosan, Latvia) for 60 min at + 37° C and 100 rpm. The supernatant was then withdrawn and centrifuged (6800 g, 20 min.) on an Avanti J-E centrifuge with a JA 14.50 rotor (Beckman Coulter, US). The supernatant was then centrifuged for 2 h at 96,200 g in an Optima L-90 K ultracentrifuge (Beckman Coulter, US) with SW28 rotor. The supernatant was removed and the pellet dissolved in 1 ml of 0.1 M Tris–HCl buffer (pH 7.0). The resulting suspension was subsequently filtered through 1.2 μm, 0.45 μm and 0.22 μm filters (MF-Millipore, US). The resulting filtrate was stored at 4° C.

### Bacteriophage amplification and purification

800 ml of Brain Heart Infusion (BHI) broth and 10 ml of an overnight culture of *Klebsiella pneumoniae* cells were mixed. In each shaking flask, cells of a specific *Klebsiella pneumoniae* strain (Kl 327, Kl 325, Kl 315 and Kl 263) were presented. The mixture was incubated on an Environmental Shaker-Incubator ES-20/60 (Biosan, Latvia) for 2 h at + 37° C and 100 rpm. Then 1 ml of bacteriophage filtrate was added to the flasks and incubated overnight. In the morning, the lysis of the bacterial culture was observed. The contents of the flasks were differentially centrifuged and the resulting was then subsequently filtered as described above. The filtrates were stored at 4° C in a refrigerator.

### Bacteriophage plaque assays

A suspension of *Klebsiella pneumoniae* cells in physiological salt solution was prepared. The concentration of cells in suspension is approximately 10^9^ cfu/ml (colony forming units per ml). Then, serial dilutions (from 10^–1^ to 10^–10^) of each of four obtained filtrates with bacteriophages in physiological salt solution were prepared. 0.2 ml of culture and 0.1 ml of a specific dilution of the bacteriophage were added to tubes with soft agar (0.6%), mixed. The resulting mixture was distributed on Petri dishes with solid agar. Incubated 24 h at a temperature of + 37 °C in a thermostat (Binder GmbH, Germany). After the incubation period, the obtained plaques were evaluated.

### Preparation of "clean lines" of bacteriophages and evaluation of plaques morphology

Single plaques with agar fragments obtained for each of the four filtrates were excised from soft agar and placed in different tubes with 1 ml of physiological salt solution. They were incubated for 10 min at room temperature. Then the extracts were taken and amplification/plaque assays steps were repeated (3 repetitions were made). The obtained filtrates were stored at 4° C in a refrigerator. After plaque assays described above, the morphology of plaques was evaluated.

### Determination of phage host range

To assess the lytic spectrum of the accumulated bacteriophage, the double-layer method was used. As a test culture, 14 strains of *Klebsiella pneumoniae* were used. A suspension of *Klebsiella pneumoniae* cells in physiological salt solution was prepared. The concentration of cells in suspension is approximately 10^9^ cfu/ml. 0.2 ml of culture were added to tubes with soft agar (0.6%), mixed, poured on Petri dish with agar and allowed to solidify. Then, the phage suspension in several dilutions was spotted on the soft agar. Petri dishes were incubated for 24 h at + 37° C in a thermostat (Binder GmbH, Germany). After incubation, the presence of lysis spots was assessed. To verify that the formation of lysis spots was caused by the lytic action of the phage, bacteriophage plaque assays were performed as described above*.* Moreover, an analysis of lysis spots on a JEM-1011 electron microscope (JEOL, Japan) was performed. For this, a fragment of the agar plate was taken from the lysis spot and placed in a 0.1 ml drop of physiological saline. Incubated for 25–30 min. After that, the agar plate was removed and electron microscopy of the droplet was carried out. The presence of bacteriophages in the test material was evaluated.

### Centrifugation in CsCl gradient

Solutions with different percentages of CsCl (50, 30, 20, 10, 5) were prepared. The solvent used was 0.05 M Tris–HCl buffer (pH 7.0). After that, the solutions were carefully added to the centrifuge tube from the SW 28 rotor, layering the gradient from a higher concentration to a lower one using a Pasteur pipette. The bacteriophage filtrate was added last. At each stage, phase uniformity was controlled, preventing their mixing. Tubes were equilibrated with a 5% CsCl solution and placed in an Optima L-90 K ultracentrifuge (Beckman Coulter, US). Then centrifuged for 2 h at 96,200 g. After centrifugation, the content of the zone with phage was carefully selected into eppendorf tubes using a pipette. The contents of the tubes were then centrifuged for 2 h at 96,200 g on an Optima L-90 K centrifuge (Beckman Coulter, US) with a SW 28 rotor. The pellet was resuspended in 0.05 M Tris–HCl buffer (pH 7.0). The resulting phage suspension was stored at 4° C in a refrigerator.

### Electron microscopy

Electron microscopy was performed using high-titer bacteriophage filtrates, purified in a CsCl density gradient. Samples were deposited on carbon-coated nitrocellulose films, stained with 1% uranyl acetate and examined in the transmission electron microscope (TEM) JEM-1011 (JEOL, Japan). Electron micrographs were taken with the Erlangshen ES500W (Gatan, US) camera. The parameters of bacteriophages were measured using ImageJ program based on images obtained using an electron microscopy. Standards based on measurements of the tail length (114 nm) of the bacteriophage T4 were used as markers. 35 particles were measured for each phage and SD (standard deviation) was calculated.

### Estimation of frequency of phage-resistant forms generation

Cells from an overnight culture were suspended in in physiological salt solution to 10^8^ cfu/ml, 0.1 ml of culture and 0.1 ml of phage filtrate initially containing 10^8^ pfu/ml (plaque forming units per ml) were added to tubes with soft agar (0.6%), mixed. The resulting mixture was distributed on Petri dishes with solid agar. Incubated 24 h at a temperature of + 37 °C in a thermostat (Binder GmbH, Germany). Afterwards, the number of resistant colonies was evaluated and the frequency of phage-resistant forms generation was calculated. The resulting resistant colonies were looped into a test tubes with 10 ml of BHI broth and mixed. The resulting suspensions was incubated for 2 h at 37 °C in a thermostat (Binder GmbH, Germany). Subsequently, mitomycin C was added to the test tubes to final concentrations of 0.2 μg/ml, 0.5 μg/ml and 2 μg/ml and they were incubated for 24 h at + 37 °C in a thermostat (Binder GmbH, Germany). The suspensions were then centrifuged (6800 g, 20 min) on an Avanti J-E centrifuge with a JA 14.50 rotor (Beckman Coulter, US), supernatants were taken into clean tubes, centrifuged for 2 h at 96,200 g in an Optima L-90 K ultracentrifuge (Beckman Coulter, US) with SW28 rotor and the sample was analyzed on electron microscope for phage presence in the suspension. In addition, initial suspension of resistant colonies was treated with chloroform, then centrifuged (6800 g, 20 min) on an Avanti J-E centrifuge with a JA 14.50 rotor (Beckman Coulter, US) and supernatants were spotted on phage-sensitive *Klebsiella pneumoniae* lawns.

### Sensitivity of phage particles to temperature and pH

In temperature and pH sensitivity assays the methods used by Jamal et al. were applied [41]. The bacteriophage filtrate was incubated for 1 h in test tubes in a water bath (GFL, Germany) at temperatures of 37, 50, 55, 60, 65 and 70 °C. Then, serial dilutions of the bacteriophage filtrate from 10^–2^ to 10^–10^ were prepared in increments of 10^2^. Phage titer was obtained by plaque assay as described above. To study pH sensitivity, tubes with meat-peptone broth (MPB) (initial pH 7.2) were prepared with pH values ​​from 3 to 13. To obtain the desired pH values, 6 M NaOH and HCl solutions were used. Filtered MPB through filters with a pore diameter of 0.22 μm (MF-Millipore, US). 1 ml of phage was added to the tubes with each pH value and incubated for 18 h at + 37° C in an incubator (Binder GmbH, Germany). The pH was monitored after the incubation period. Same incubation period for pH sensitivity assay was also used by Soleimani Sasani and Eftekhar [42]. Then, serial dilutions of the solution with phages from 10^–1^ to 10^–10^ were prepared and plaque assay done as described above to obtain phage titer.

### Phage adsorption rate

Cells from an overnight culture were suspended in BHI broth to 10^9^ cfu/ml. 5 ml of bacterial suspension and phage suspension diluted to 10^7^ pfu/ml were incubated in 45 ml of BHI broth on Environmental Shaker-Incubator ES-20/60 (Biosan, Latvia) at + 37 °C and 100 rpm for 5 min. After that the supernatant was filtered through 0.22 μm pore filter and the free phages were enumerated using the plaque assay described above. The reduction in phage titer was the number of phages adsorbed to the cells. Bacteriophage filtrate was used as a control. A decrease in titer in the control was not observed. The adsorption constant was calculated by the following formula:

$$k = \left( {\frac{ - 1}{{Bt}}} \right) \times \ln \left( {\frac{P}{{P_{0} }}} \right)$$

P–concentration of free phage per ml, P_0_–initial concentration of phage, B–initial concentration of bacteria, k–adsorption rate constant (ml/min), t—time (min).

### One-step growth curve

1 ml of BHI broth, a cell suspension with a final concentration of 10^9^ cfu/ml and a bacteriophage filtrate with a final concentration of 10^7^ pfu/ml were mixed. The mixture was incubated at 37 °C for 8 min in a thermostat (Binder GmbH, Germany) and then centrifuged for 2 min at 4800 g on Centrifuge 5424 (Eppendorf, Germany). The supernatant was removed and the pellet resuspended in 100 ml of BHI broth. Aliquots of 0.5 ml of the resulting suspension were taken every 5 min for 80 min and the bacteriophage titer was assessed using plaque assay described above. The latency period was defined as the the interval between adsorption of the phage to the host cell and release of phage progeny. The burst size of the phage was expressed as the ratio of the final count of released phage particles to the number of infected bacterial cells during latent period.

### Phage propagation in liquid nutrient medium and evaluation of the bacteriophage titer

Cells from an overnight culture were suspended in BHI broth to 10^9^ cfu/ml. 5 ml of bacterial suspension containing 10^9^ cfu/ml and phage diluted to 10^6^ pfu/ml were incubated in 45 ml of BHI broth on Environmental Shaker-Incubator ES-20/60 (Biosan, Latvia) at + 37 °C and 100 rpm for 18 h. As a control one flask had no phage added to the *Klebsiella* culture. Flasks were incubated for 18 h at + 37 °C and 100 rpm. After that, the phage titre in the experimental system was evaluated using plaque assays described above. Incubated for 24 h at + 37 °C in a thermostat (Binder GmbH, Germany). After incubation, the phage titer was evaluated.

### Isolation and restriction digestion of phage DNA

Phage genomic DNA was extracted from high-titre bacteriophage filtrates, purified in a CsCl density gradient. An ethylenediaminetetraacetic acid (EDTA) solution was added to 0.45 ml of the bacteriophage filtrate to a final concentration of 25 mM. Then, proteinase K was added to a final concentration of 100 μg/ml and incubated at + 50 °C for 2 h. After that, sodium dodecyl sulfate (SDS) was added to a concentration of 0.5% and incubated at + 55 °C for 2 h. Then the mixture was heated for 20 min at + 65 °C to inactivate the enzymes. Next, a double extraction with chloroform was performed. 0.5 ml of isopropyl alcohol was added to the aqueous phase, after which the DNA was extracted onto a glass rod. After washing three times in a 70% ethanol solution, the DNA was dried for 5 min on air, and then dissolved in 0.4 ml of TE buffer (pH 8.0). The quantity and quality of the extracted DNA was monitored by analysis on a NanoDrop ND-1000 spectrophotometer (ThermoFisher, US).

Phage DNA was digested with restriction enzymes according to the manufacturer's protocol for 90 min at + 37 °C (at + 30 °C for SmaI) in the buffer and conditions appropriate for restriction enzyme (in 50 μl of the reaction: 5 units of the enzyme, 1 μg of DNA, and the volume of distilled water). The following restriction endonucleases were used: HindIII, HinfI, HaeIII, SspI, BamHI, EcoRV, NotI, EcoRI, KpnI, MspI, VspI, NdeI, BgII, BgIII, PvuI, SmaI. To analyze the results, electrophoresis was performed on a 1% agarose gel at 180–200 V for 1 h at room temperature on a Sub-cell Model 92 Cell (Biorad). 15–20 μl (1–2 μl of buffer for application to 5 μl of reaction) were added to the wells. Lambda DNA/HindIII (Thermo Scientific, US) was used as markers.

Visualization of the results was carried out after staining agarose gels for 20 min in a solution of ethidium bromide (1 μg/ml). To document the results, the DOCPRINT system (Vilber Lourmat, France) was used. The obtained restriction profiles were compared with those predicted in silico using the RestrictionMapper program (restrictionmapper.org).

### Genome sequencing, assembly and annotation

Phage DNA was extracted by chloroform extraction [43]. The DNA library was constructed using a Nextera DNA Library Preparation Kit (Illumina, San Diego, CA) and sequenced with an Illumina HiSeq T1500 sequencer, resulting in approximately 1 million 2 × 250 paired-end reads. The quality control and primary processing was performed using FASTQC and trimmomatic (HEADCROP:20, SLIDINGWINDOW:3:24, MINLEN:200, CROP:200) [44]. Coverage was normalized to × 50 using BBNorm [45]. De novo assembly was performed using MIRA assembler [46] version 4.9.6. Contig completion was confirmed by comparison to closely related genomes known to be complete (accession numbers are presented in Table 5). Open reading frames search was performed using MetaProdigal 2.6 [47]. Annotation was performed using all peer-reviewed phage and bacterial proteins from UniProt (https://www.uniprot.org) and all proteins from databases of determinants of antimicrobial resistance and bacterial virulence factors: VFDB [48], CARD [49], ARG-ANNOT [50] and Resfinder [51]. The remaining ORFs were annotated with hmmscan application (minimum e-value 0.001) [52], using all bacterial HMM profiles from the Pfam database (https://pfam.xfam.org). tRNA was predicted using tRNAscan-SE 2.0 [53]. All analyses, except those indicated, were performed using default parameters. BLASTn (https://blast.ncbi.nlm.nih.gov/Blast.cgi) was used to search for similarity with other bacteriophages, and to calculate average nucleotide identity and query coverage.

The complete genome sequences of *Klebsiella pneumoniae* phages vB_KpnS_FZ10, vB_KpnP_FZ12, vB_KpnM_FZ14 and vB_KpnS_FZ41 have been deposited in GenBank under the accession numbers MK521904, MK521905, MK521906 and MK521907, respectively. Raw Illumina reads are available on NCBI SRA under accession numbers SRR10037530, SRR10037529, SRR10037528 and SRR10037527, respectively. The associated BioProject accession number is PRJNA562287. GenomeVx tool [54] was used for complete genome visualization. Taxonomic identification was made by GenBank (https://www.ncbi.nlm.nih.gov/genbank/) based on the phylogenetic classification scheme used in the NCBI Taxonomy Database (https://www.ncbi.nlm.nih.gov/Taxonomy).

## Results

### Isolation and morphology

**Fig. 1:**
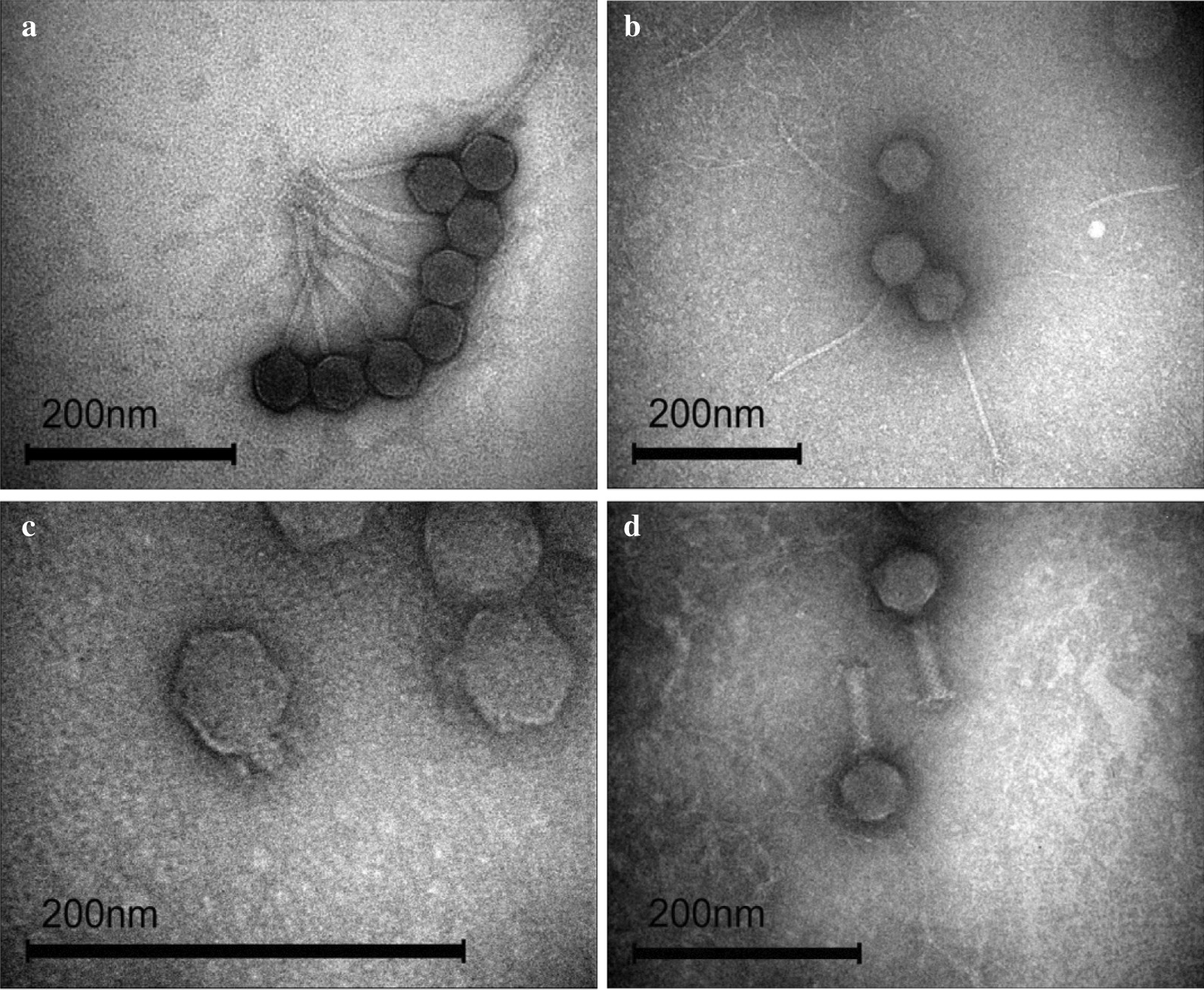
Electron micrographs of bacteriophages vB_KpnS_FZ10 (**a**), vB_KpnS_FZ41 (**b**), vB_KpnP_FZ12 (**c**), and vB_KpnM_FZ14 (**d**). Contrasting with 1% solution of uranyl acetate in distillated water. Magnification x250k for micrograph A, x200k for B, x600k for C and x300k for D

Four bacteriophages were studied in the present work. Coastal bottom sediments of wastewater and freshwater bodies were selected as a source for the isolation of bacteriophages. According to published data, they contain the largest number of viral particles [55–58]. It has been shown that phages are often adsorbed on inorganic colloids due to Van der Waals forces [59]. Our laboratory uses the method of isolation using solutions with high ionic strength to desorb bacteriophages from colloidal particles. Despite the fact, that salt enhances the Van der Waals interactions, it is also able to destroy hydrogen bonds, and contributes to a change in the charge of colloidal particles under the action of positively charged ions. That is why we performed isolation using a solution of 1 M NaCl. This method leads to increase in number of phages in the sample and is recommended for isolating other viruses. Phages were isolated from water samples taken from waste-water treatment plants near Moscow, Russia. Their morphological features were examined by transmission electron microscopy (Fig. 1).

Phages were named vB_KpnM_FZ14, vB_KpnS_FZ10, vB_KpnS_FZ41 and vB_KpnP_FZ12, according to the naming system proposed by Kropinski et al.[60]. vB_KpnM_FZ14 has isometric capsid and long contractile tail and can be assigned to the *Myoviridae* family based on morphological characteristics, vB_KpnS_FZ10 and vB_KpnS_FZ41 have isometric capsids and long non-contractile tails and can be assigned to the *Siphoviridae* family based on morphological characteristics, vB_KpnP_FZ12 has isometric capsid and short non-contractile tail and can be assigned to the *Podoviridae* family based on morphological characteristics. Morphological features and plaque morphology are described in Table 1.

**Table 1:** Morphological features and plaque morphology of isolated *Klebsiella pneumoniae* bacteriophages

Morphology	Phage vB_KpnS_FZ10	Phage vB_KpnS_FZ41	Phage vB_KpnP_FZ12	Phage vB_KpnM_FZ14
	Isometric capsid, long non-contractile tail	Isometric capsid, long non-contractile tail	Isometric capsid, short non-contractile tail	Isometric capsid, long contractile tail
Average head diameter (± SD), nm	61 ± 2	71 ± 5	49 ± 3	55 ± 3
Tail length (± SD), nm	158 ± 13	227 ± 25	-	79 ± 5
Clear plaque size, mm	1–2	0.3–0.5	0.7–2	0.7–1.5
Halo	+	-	+	+

Head diameter is calculated for isometric capsids. All measurements were made with ImageJ program, 35 particles were measured for each phage and standard deviation was calculated (± SD). Clear plaque size is the calculation of transparent plaque zone diameter, presence of halo is indicated with “ + ”, absence with “- “

**Fig. 2:**
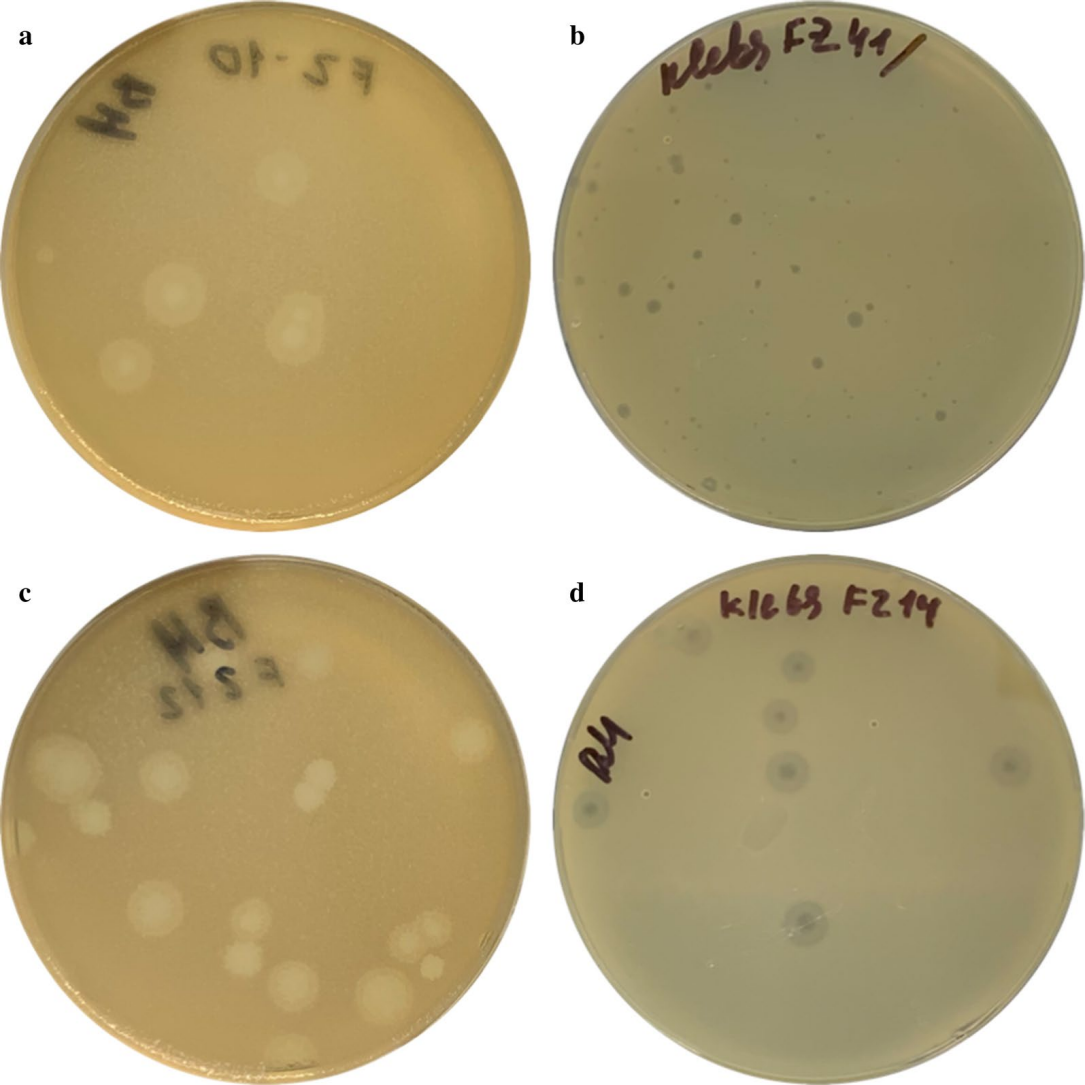
Photographs of the plaques formed by bacteriophages vB_KpnS_FZ10 (**a**), vB_KpnS_FZ41 (**b**), vB_KpnP_FZ12 (**c**), and vB_KpnM_FZ14 (**d**). acteriophages vB_KpnS_FZ10, vB_KpnP_FZ12 and vB_KpnM_FZ14 form plaques with a halo. Bacteriophage vB_KpnS_FZ41 forms completely transparent lysis zones

Evaluation of the plaque morphology showed that the bacteriophage vB_KpnS_FZ41 from the *Siphoviridae* family forms completely transparent lysis zones. All other studied phages (vB_KpnS_FZ10, vB_KpnP_FZ12 and vB_KpnM_FZ14) form a halo. Such plaques always had a central transparent part, and the size of the halo increased with incubation time. Plaque morphology is shown in Fig. 2.

### Determination of phage host range

The lytic activity of each bacteriophage was examined on 14 clinical strains of *Klebsiella pneumoniae*, obtained from different hospitals in Moscow region, Russia (Table 2). In addition, lytic activity of different combinations of phages was tested. All tests were done in three repetitions.

**Table 2:** Lytic spectra of the bacteriophages vB_KpnS_FZ10, vB_KpnS_FZ41, vB_KpnP_FZ12, and vB_KpnM_FZ14

*Klebsiella pneumoniae* strain	FZ10	FZ41	FZ12	FZ14	Combination (All)	Combination (FZ10 + FZ12 + FZ14)	Combination (FZ12 + FZ41)
Kl A1265	-	-	+	-	+	+	+
Kl 43,816	+	-	+	+	+	+	+
Kl 315	+	-	+	+	+	+	+
Kl 3–53	+	-	+	+	+	+	+
Kl 610	-	-	+	-	+	+	+
Kl 7880	+	-	+	-	+	+	+
Kl 327	+	-	+	-	+	+	+
Kl 12–1	+	+	+	-	+	+	+
Kl 27–89	-	+	-	-	+	-	+
Kl 293	-	+	-	-	+	-	+
Kl 263	-	-	+	+	+	+	+
Kl 3273	+	-	+	-	+	+	+
Kl T-14	+	-	-	-	+	+	-
Kl 325	-	+	-	-	+	-	+
Total « + »	8/14	4/14	10/14	4/14	14/14	11/14	13/14

" + " indicates the presence of sensitivity of a bacterial strain to the action of a bacteriophage. "-" indicates the absence of sensitivity of a bacterial strain to the action of a bacteriophage. Identical results were obtained in 3 repetitions

Bacteriophages vB_KpnS_FZ10 and vB_KpnP_FZ12 were active against high percentage of bacterial strains (57% and 71%, respectively). While the lytic activity of the phages vB_KpnS_FZ41 and vB_KpnM_FZ14 was lower (29% each). However, overlapping lytic spectra of all 4 viruses allows their combination to be effective against all studied bacterial strains. Additionally, plaque assays were performed to confirm that the lysis spots were formed due to the lytic action of bacteriophages (Table 3).

**Table 3:** Titers of bacteriophages vB_KpnS_FZ10, vB_KpnS_FZ41, vB_KpnP_FZ12, and vB_KpnM_FZ14 obtained on different *Klebsiella pneumoniae* strains

*Klebsiella pneumoniae* strain	FZ10	FZ41	FZ12	FZ14	Combination (all)
Kl A1265	-	-	$3 \times 10^{7}$	-	$3.6 \times 10^{6}$
Kl 43,816	$1 \times 10^{7}$	-	$4 \times 10^{7}$	$4.6 \times 10^{8}$	$2 \times 10^{8}$
Kl 315	$8 \times 10^{6}$	-	$6 \times 10^{9}$	$4 \times 10^{9}$	$1 \times 10^{9}$
Kl 3–53	$5.2 \times 10^{6}$	-	$1 \times 10^{8}$	$4 \times 10^{8}$	$4 \times 10^{8}$
Kl 610	-	-	$1 \times 10^{7}$	-	$2 \times 10^{6}$
Kl 7880	$4 \times 10^{8}$	-	$8 \times 10^{6}$	-	$2 \times 10^{8}$
Kl 327	$3.2 \times 10^{8}$	-	$2 \times 10^{6}$	-	$1 \times 10^{8}$
Kl 12–1	$4 \times 10^{7}$	$1 \times 10^{3}$	$1.4 \times 10^{7}$	-	$1 \times 10^{7}$
Kl 27–89	-	$2 \times 10^{6}$	-	-	$1.8 \times 10^{6}$
Kl 293	-	$4.4 \times 10^{7}$	-	-	$4 \times 10^{6}$
Kl 263	-	-	$6 \times 10^{8}$	$1 \times 10^{8}$	$1 \times 10^{8}$
Kl 3273	$4 \times 10^{8}$	-	$2.2 \times 10^{8}$	-	$2 \times 10^{7}$
Kl T-14	$3.6 \times 10^{8}$	-	-	-	$4 \times 10^{8}$
Kl 325	-	$8 \times 10^{8}$	-	-	$2.2 \times 10^{8}$

Titers were evaluated after a series of plaque assays. "-" indicates the absence of sensitivity of a bacterial strain to the action of a bacteriophage

### Biophysical stability

**Fig. 3:**
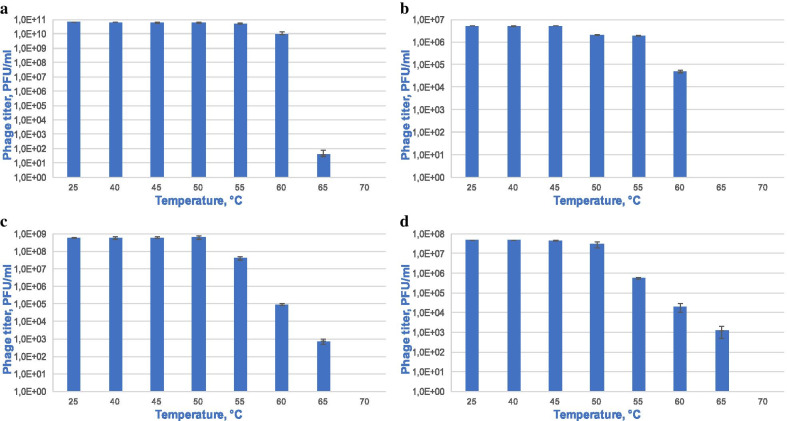
Bacteriophages vB_KpnS_FZ10 (**a**), vB_KpnP_FZ12 (**b**), vB_KpnM_FZ14 (**c**), and vB_KpnS_FZ41 (**d**) temperature stability. Incubation for 1 h at temperatures of 37, 50, 55, 60, 65 and 70 °C. Results are based on three repetitions. The deviation from the average value is indicated on the graph

To study temperature stability, the following temperatures were selected: 25, 40, 45, 50, 55, 60, 65, and 70° C. To assess stability, the titer of the virus was analyzed after an hour of incubation in Tris–HCl buffer. The data is shown in Fig. 3.

At 65° C, a significant drop in titer of bacteriophages vB_KpnS_FZ10, vB_KpnS_FZ41 and vB_KpnM_FZ14 is observed, and phage vB_KpnP_FZ12 is completely inactivated.

**Fig. 4:**
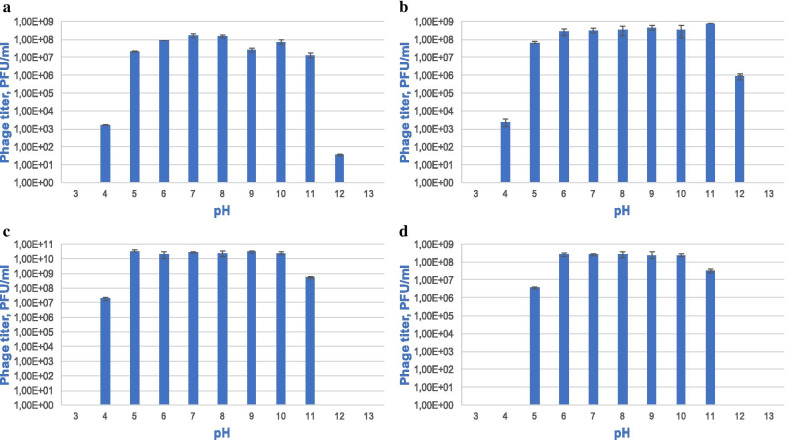
Bacteriophages vB_KpnS_FZ10 (**a**), vB_KpnP_FZ12 (**b**), vB_KpnM_FZ14 (**c**), and vB_KpnS_FZ41 (**d**) pH stability. Incubation for 18 h at pH values from 3 to 13. Results are based on three repetitions. The deviation from the average value is indicated on the graph

For studying pH stability, the following pH values ​​were chosen: 3, 4, 5, 6, 7, 8, 9, 10, 11, 12, 13. To assess stability, the titer of the virus was analyzed after incubation for 18 h. All bacteriophages were stable after incubation at pH values ​​from 5 to 11. At pH 4, a decrease in the titer of bacteriophages vB_KpnS_FZ10, vB_KpnP_FZ12 and vB_KpnM_FZ14 was observed, and vB_KpnS_FZ41 was completely inactivated. At pH 12, vB_KpnP_FZ12 turned out to be the most stable, a significant titer drop was detected for phage vB_KpnS_FZ10, bacteriophages vB_KpnM_FZ14 and vB_KpnS_FZ41 were completely inactivated. All data is shown in Fig. 4.

### The adsorption rate

The experiment showed that in 5 min, from 79% (vB_KpnS_FZ41) to 93% (vB_KpnP_FZ12) of viral particles are adsorbed on the host cell, depending on the phage (Table 4).

**Table 4:** Adsorption dynamics of bacteriophages vB_KpnS_FZ10, vB_KpnS_FZ41, vB_KpnP_FZ12, and vB_KpnM_FZ14 on *Klebsiella pneumoniae* cells

Bacteriophage	Bacteriophage titer, pfu/ml			
	Incubation time, 0 min	Incubation time, 5 min	% of adsorbed phages	Adsorption constant, *k*
vB_KpnS_FZ10	$2.8 \times 10^{7} \pm 8.2 \times 10^{6}$	$3.2 \times 10^{6} \pm 1.6 \times 10^{5}$	$89$	$4.3 \times 10^{ - 9}$
vB_KpnS_FZ41	$1.1 \times 10^{7} \pm 1.2 \times 10^{6}$	$2.3 \times 10^{6} \pm 0.8 \times 10^{5}$	$79$	$3.1 \times 10^{ - 9}$
vB_KpnP_FZ12	$3.9 \times 10^{7} \pm 1.4 \times 10^{6}$	$2.7 \times 10^{6} \pm 1 \times 10^{5}$	$93$	$5.3 \times 10^{ - 9}$
vB_KpnM_FZ14	$2.3 \times 10^{7} \pm 2 \times 10^{6}$	$3 \times 10^{6} \pm 1.6 \times 10^{5}$	$87$	$4 \times 10^{ - 9}$

Dependence of the titer of bacteriophages not adsorbed on *Klebsiella pneumoniae* cells on the incubation time in the phage-cell system is indicated. The average value (± SD) is calculated based on the results of three repetitions. The reduction in phage titer was the number of phages adsorbed on the cells. The percentage of viruses adsorbed on cells and adsorption constant are calculated

### One-step growth

**Fig. 5:**
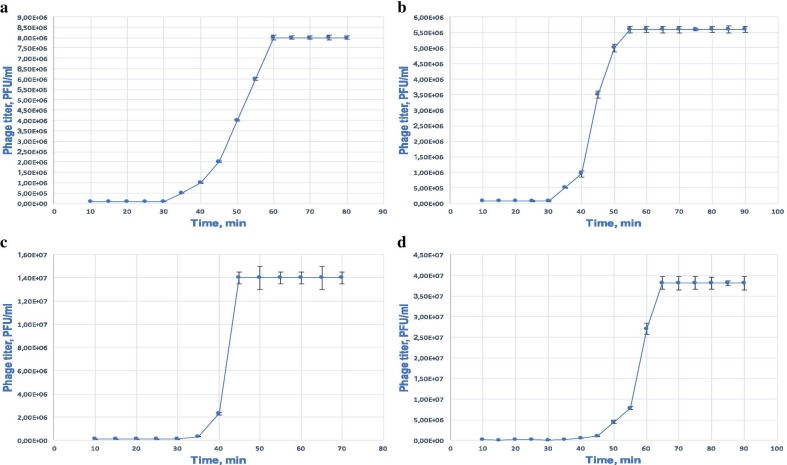
One-step growth curves of bacteriophages vB_KpnS_FZ10 (**a**), vB_KpnP_FZ12 (**b**), vB_KpnM_FZ14 (**c**) and vB_KpnS_FZ41 (**d**). The dependence of the bacteriophage titer on the incubation time in the phage-cell system with *Klebsiella pneumoniae* cells is shown. Results are based on three repetitions. The deviation from the average value is indicated on the graph

Based on the one-step growth experiment, latent period and burst size for each phage were calculated (Fig. 5).

The latent period was 30 min for the phages vB_KpnS_FZ10, vB_KpnP_FZ12 and vB_KpnM_FZ14, and for vB_KpnS_FZ41 it was 35 min. The burst size was approximately 80 particles per bacterial cell for vB_KpnS_FZ10 (80 ± 2) and vB_KpnP_FZ12 (80 ± 7) and 120 particles per bacterial cell for vB_KpnS_FZ41 (118 ± 3) and vB_KpnM_FZ14 (120 ± 5).

### Estimation of the frequency of phage-resistant forms generation

The frequency of generation of phage-resistant forms was examined for each isolated bacteriophage and for a combination of phages vB_KpnS_FZ10, vB_KpnP_FZ12 and vB_KpnM_FZ14 using the following formula:

$$F = \frac{n}{N}$$

n—the average number of phage-resistant colonies after incubation, pcs. N—initial concentration of bacterial cells in suspension, cfu/ml.

Since our bacterial collection does not contain a strain that is sensitive to all 4 phages at once, the cocktail for establishing the frequency of phage-resistant forms generation was composed of 3 bacteriophages. Experiment was conducted in 3 repetitions.

The following data was obtained:

$${\text{Bacteriophage vB}}\_{\text{KpnS}}\_{\text{FZ}}10{ } - { }F = \frac{8 \pm 3}{{2.3 \times 10^{7} }} = \left( {3.5 \pm 1.3} \right) \times 10^{ - 7} \approx 4 \times 10^{ - 7}$$

• Bacteriophage vB_KpnS_FZ41—$$F = \frac{90 \pm 16}{{10^{8} }} = \left( {9 \pm 1.6} \right) \times 10^{ - 7} \approx 9 \times 10^{ - 7}$$
• Bacteriophage vB_KpnP_FZ12—$$F = \frac{50 \pm 10}{{10^{8} }} = \left( {5 \pm 1} \right) \times 10^{ - 7} \approx 5 \times 10^{ - 7}$$
• Bacteriophage vB_KpnM_FZ14—$$F = \frac{72 \pm 9}{{10^{8} }} = \left( {7.2 \pm 0.9} \right) \times 10^{ - 7} \approx 7 \times 10^{ - 7}$$
• Phage combination (FZ10 + FZ12 + FZ14)—$F = \frac{0}{{10^{8} }} = 0$

**Fig. 6:**
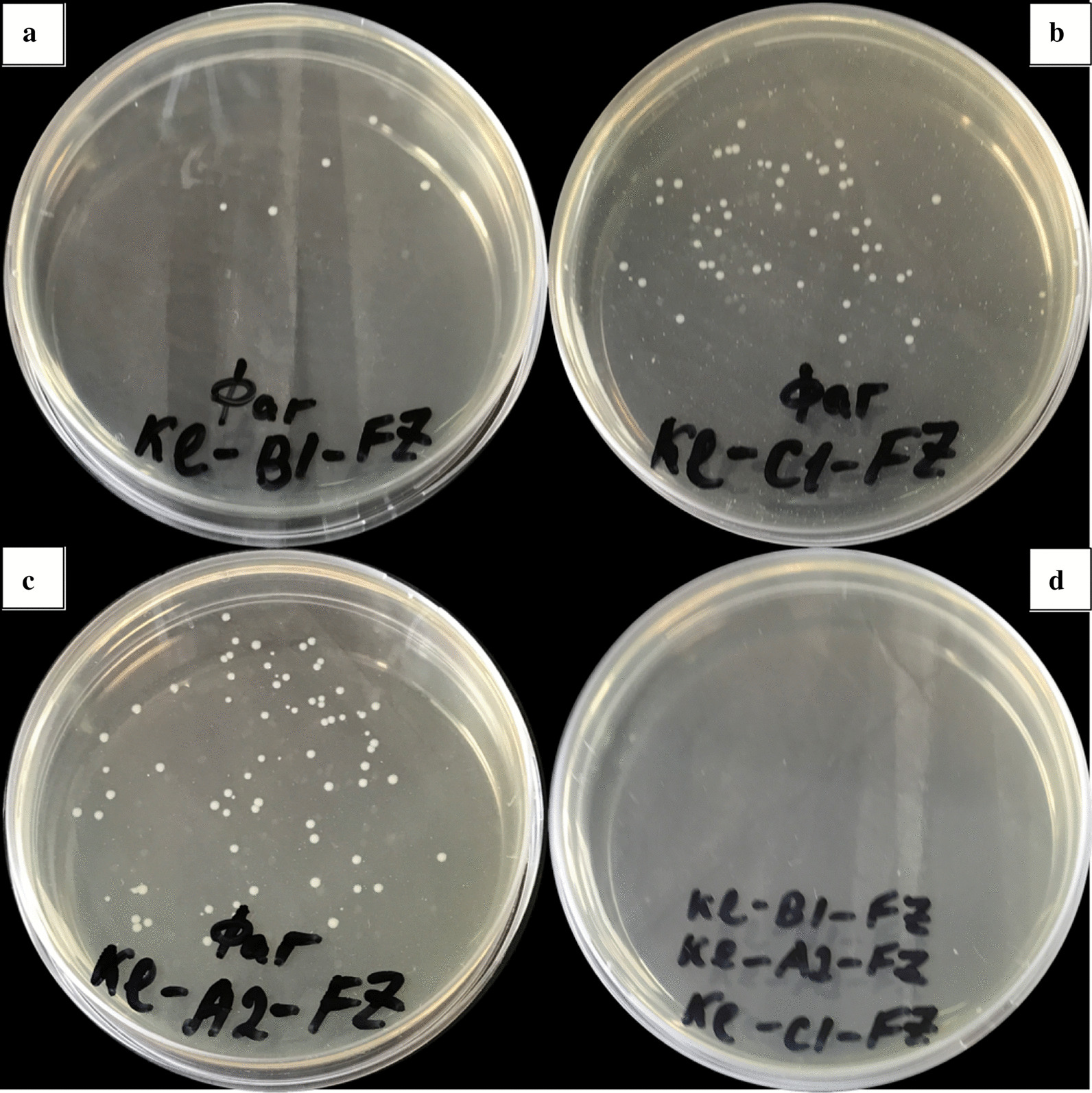
Photographs of the phage-resistant *Klebsiella pneumoniae* colonies formed after incubation with bacteriophages vB_KpnS_FZ10 (**a**), vB_KpnP_FZ12 (**b**), vB_KpnM_FZ14 (**c**) and a cocktail of 3 phages. Phage-resistant *Klebsiella pneumoniae* colonies were formed after incubation with single phage preparation. After incubation with a cocktail of all 3 bacteriophages no resistant colonies were observed. Identical results were obtained in 3 repetitions

After incubation with a cocktail of 3 bacteriophages, no phage-resistant colonies were found. Photographs of the phage-resistant colonies formed after incubation with single phages and with a cocktail of 3 bacteriophages are shown in Fig. 6.

Spot tests of the chloroform-treated suspensions of the phage-resistant colonies have not revealed the presence of prophages. Moreover, phages were not observed after TEM screening of the mitomycin C treated material from the obtained phage-resistant colonies. Thus, under the given experimental conditions, it was not possible to induce prophage from the phage-resistant culture of *Klebsiella pneumoniae*.

### Phage propagation in liquid nutrient medium and evaluation of the bacteriophage titer

Flasks with a specific culture of *Klebsiella pneumoniae* and each bacteriophage were incubated for 18 h at + 37 °C and 100 rpm. One flask had no phage added as a control. After that, the phage titer in the experimental system was evaluated using plaque assays. After 18 h of incubation, growth of the bacterial cells in the medium was observed in a flask without phage. The medium in the flasks with phages remained transparent. The average concentration (± SD) of propagated phages according to 3 plaque assays was:

- vB_KpnS_FZ10 – $2.5 \times 10^{10} \pm 3.3 \times 10^{9}$ pfu/ml;
- vB_KpnS_FZ41 – $2.5 \times 10^{9} \pm 4.2 \times 10^{8}$ pfu/ml;
- vB_KpnP_FZ12 – $5 \times 10^{9} \pm 1.6 \times 10^{9}$ pfu/ml;
- vB_KpnM_FZ14 – $9 \times 10^{9} \pm 2.4 \times 10^{9}$ pfu/ml.

### Restriction analysis of phage DNA

DNA of phage vB_KpnS_FZ10 is hydrolyzed by restriction enzymes HaeIII, SspI, BamHI, EcoRV, EcoRI and is not sensitive to restriction enzymes HindIII, SmaI, NotI and KpnI. For NotI, one cut site in phage genome was predicted in silico, but phage DNA was not sensitive to this enzyme. For the rest of restriction enzymes, the results obtained are in line with the predicted ones.

DNA of phage vB_KpnS_FZ41 is hydrolyzed by restriction enzymes EcoRI (O), VspI, NdeI, BgII, BgIII, EcoRV, PvuI, KpnI, EcoRI (RI), HinfI, BamHI and is not sensitive to restriction enzyme MspI. For MspI, 313 cut sites in phage genome were predicted in silico, but phage DNA was not sensitive to this enzyme. For the rest of restriction enzymes, the results obtained are in line with the predicted ones.

DNA of phage vB_KpnP_FZ12 is hydrolyzed by restriction enzymes HinfI, EcoRV, MspI, KpnI and is not sensitive to restriction enzymes HindIII, SspI, BamHI and EcoRI. The restriction profile is fully consistent with the predicted in silico.

DNA of phage vB_KpnM_FZ14 is hydrolyzed by restriction enzymes HindIII, EcoRV, EcoRI, SmaI, SalI, BamHI, KpnI, DraI, HinfI, MspI and is not sensitive to restriction enzyme NdeI. For NdeI, 9 cut sites in phage genome were predicted in silico, but phage DNA was not sensitive to this enzyme. For the rest of restriction enzymes, the results obtained are in line with the predicted ones.

**Fig. 7:**
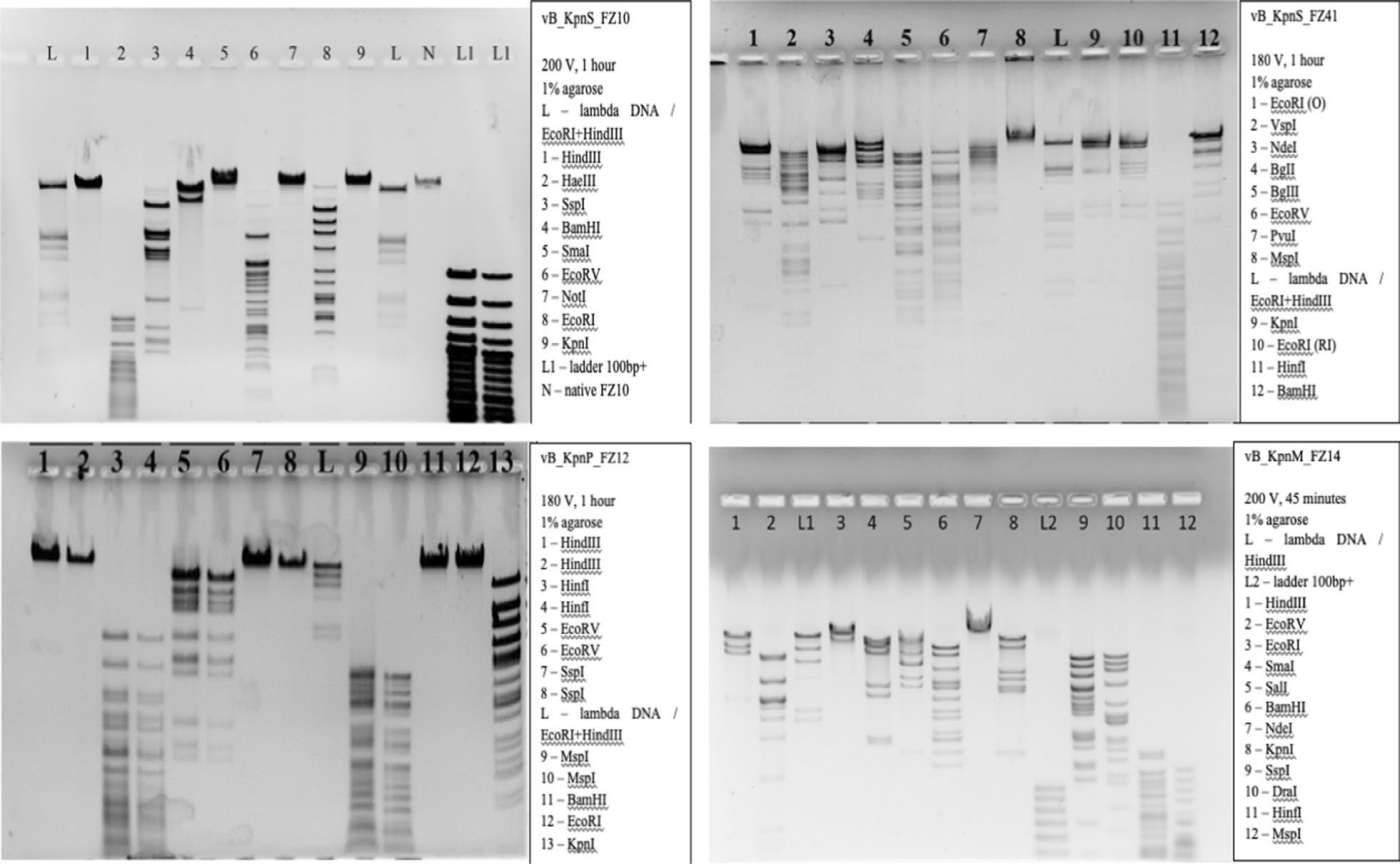
DNA electrophoresis of bacteriophages vB_KpnS_FZ10, vB_KpnS_FZ41, vB_KpnP_FZ12, and vB_KpnM_FZ14 after treatment with restriction enzymes. Enzymes, markers and electrophoresis parameters are indicated in the appendix to the figure

The results are presented in Fig. 7.

### Complete genome sequence

All bacteriophages contain double-stranded DNA. Genomic data showed that isolated phages do not carry antibiotic resistance, virulence or lysogenic genes. It was found that the bacteriophage vB_KpnS_FZ10 encodes its own adenine methyltransferase (QCG76428.1) and cytosine methyltransferase (QCG76436.1), which is a way of protecting against host restriction-modification systems. And vB_KpnS_FZ41 encodes its own tRNAs (a total of 25 tRNAs were predicted).

**Fig. 8:**
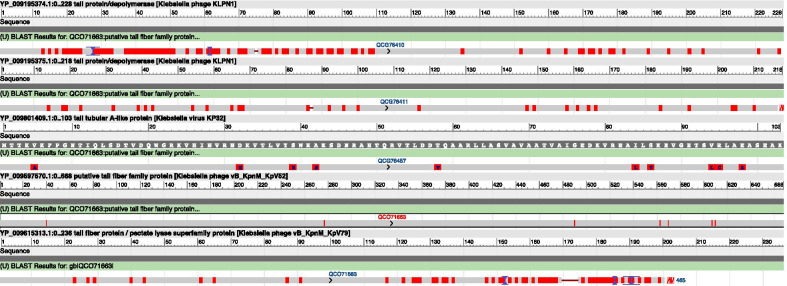
Bacteriophages vB_KpnS_FZ10, vB_KpnP_FZ12 and vB_KpnM_FZ14 polysaccharide depolymerases sequences alignment to the depolymerases of related phages. The alignment to the sequences of studied proteins with confirmed polysaccharide degrading activity was performed using BLASTp (https://blast.ncbi.nlm.nih.gov/Blast.cgi). ORFs №14 (QCG76410.1) and №15 (QCG76411.1) of vB_KpnS_FZ10 were aligned to ORFs №34 and №35 of bacteriophage KLPN1 (NC_028760). ORF №19 (QCG76457.1) of vB_KpnP_FZ12 was aligned to ORF №31 (YP_003347549.1) of bacteriophage KP32 (NC_013647). ORF №8 (QCO71663.1) of vB_KpnM_FZ14 was aligned to ORFs №42 (YP_009597570.1; YP_009615313.1) of bacteriophages KpV52 (NC_041900), KpV79 (NC_042041)

Bacteriophages vB_KpnS_FZ10, vB_KpnP_FZ12 and vB_KpnM_FZ14 encode polysaccharide depolymerases, which are involved in the destruction of biofilms and capsules. The search for the homology of encoded proteins with the studied polysaccharide depolymerases of related phages showed that ORFs №14 (QCG76410.1) and №15 (QCG76411.1) of vB_KpnS_FZ10 are homologous with ORFs №34 and №35 of bacteriophage KLPN1 (NC_028760), whose protein products (tail proteins) have endosialidase domain and were confirmed to have polysaccharide degrading activity [61]. ORF №19 (QCG76457.1) of vB_KpnP_FZ12 is homologous with ORF №31 (YP_003347549.1) of bacteriophage KP32 (NC_013647), whose protein product (Tail tubular protein A) have peptidoglycan hydrolase domain and was confirmed to have polysaccharide degrading activity [62]. ORF №8 (QCO71663.1) of vB_KpnM_FZ14 is homologous with ORFs №42 (YP_009597570.1; YP_009615313.1) of bacteriophages KpV52 (NC_041900), KpV79 (NC_042041), whose protein product (putative tail fiber family protein) have hyaluronate lyase and pectate lyase 3 domain and was confirmed to have polysaccharide degrading activity [63]. Thus, the formation of halo correlates with the presence of putative depolymerase proteins. The alignment of the sequences of all the listed proteins was made with BLASTp (https://blast.ncbi.nlm.nih.gov/Blast.cgi). The results are presented in Fig. 8.

Details on genome data are presented in Table 5. BLASTn (https://blast.ncbi.nlm.nih.gov/Blast.cgi) was used to calculate average nucleotide identity and query coverage. Taxonomic identification was made by GenBank (https://www.ncbi.nlm.nih.gov/genbank/) based on the phylogenetic classification scheme used in the NCBI Taxonomy Database (https://www.ncbi.nlm.nih.gov/Taxonomy).

**Table 5:** Genome data of bacteriophages vB_KpnS_FZ10, vB_KpnP_FZ12, vB_KpnM_FZ14, and vB_KpnS_FZ41

Bacteriophage	*Klebsiella pneumoniae* strain, NCBI ID	Genome size, bp	G + C content, %	Coverage	№ of ORFs	Taxonomic identification (Family, genus)	Related viruses	Identity and query coverage
vB_KpnS_FZ10	*Klebsiella pneumoniae* RV_BA_03_B LBK, 573	50,381	50.66	65.38	42	*Drexlerviridae*, *Webervirus*	*Klebsiella* phage NJR15, (MH633487)	96.71% (94% query coverage)
vB_KpnP_FZ12	*Klebsiella pneumoniae ssp pneumoniae* DSM 30104 T HAM, 72,407	39,519	53.06	71.03	43	*Autographiviridae, Przondovirus*	*Klebsiella* phage vB_KpnP_KpV763 (KX591654)	94.43% (93% query coverage)
vB_KpnM_FZ14	*Klebsiella pneumoniae ssp pneumoniae* 9295_1 CHB, 72,407	49,370	48.58	71.60	35	*Myoviridae, Jedunavirus*	*Klebsiella* phage vB_KpnM_KpV52 (KX237516)	96.58% (79% query coverage)
vB_KpnS_FZ41	*Klebsiella pneumoniae ssp pneumoniae* DSM 30104 T HAM, 72,407	106,104	45.22	72.25	103	*Demerecviridae, Sugarlandviru*s	*Klebsiella* phages vB_Kpn_IME260 and Sugarland (KX845404, NC_042093)	96.77% and 97.52% (93% and 89% query coverage)

*Klebsiella pneumoniae* strain used for propagation, genome size (bp), G + C content (%), average coverage and number of open reading frames (ORFs) indicated for each phage; “Related virus” refers to a top hit from NCBI BLAST, average nucleotide identity and query coverage were calculated by BLASTn

**Fig. 9:**
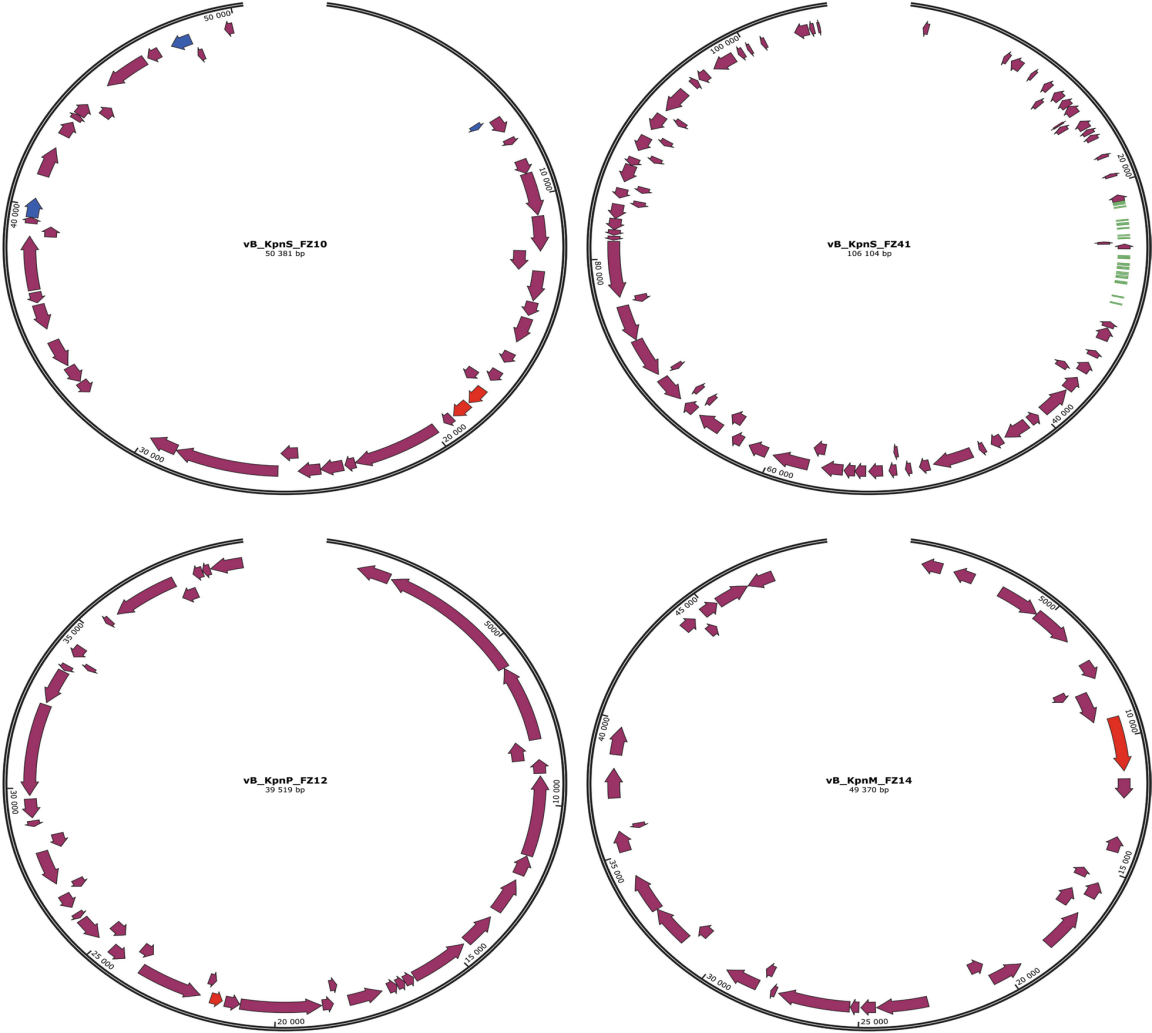
Genome maps of bacteriophages vB_KpnS_FZ10, vB_KpnS_FZ41, vB_KpnP_FZ12 and vB_KpnM_FZ14. Annotation was performed using all peer-reviewed phage and bacterial proteins from UniProt (https://www.uniprot.org) and all proteins from databases of determinants of antimicrobial resistance and bacterial virulence factors: VFDB [48], CARD [49], ARG-ANNOT [50] and Resfinder [51]. The remaining ORFs were annotated with hmmscan application (minimum e-value 0.001) [52], using all bacterial HMM profiles from the Pfam database (https://pfam.xfam.org). tRNA was predicted using tRNAscan-SE 2.0 [53]

Based on the obtained data, genome maps of studied bacteriophages were constructed (Fig. 9).

Genome maps are showing the genes within the genome of bacteriophages vB_KpnS_FZ10, vB_KpnP_FZ12, vB_KpnM_FZ14, and vB_KpnS_FZ41. The frames encoding methyltransferases are marked in blue, polysaccharide depolymerases—in red, tRNAs- in green, and all others—in violet. More detailed genome maps are presented in Additional File 2.

## Discussion

As covered in the introduction, bacteriophages are a promising tool in the fight against antibiotic-resistant bacteria [8]. However, this formulation should not be considered as a proposal to completely abandon the use of antibiotic drugs. It was shown that combined use of phages and antimicrobials can restore sensitivity of bacteria to antibiotics, because during mutagenesis and selection towards phage-resistant forms of bacteria, antibiotic resistance mechanisms are lost [64]. Moreover, the effect of phage polysaccharide depolymerases [32, 33] can facilitate drug delivery due to degradation of biofilms. Therefore, phage preparations should be considered as an option of first choice and a way to reduce the amount and dose of antimicrobial agents consumed.

It is known that one of the most common causes of nosocomial infections, including pneumonia and infections of the genitourinary system, are bacteria of the *Klebsiella pneumoniae* species [11]. The group of ESKAPE microorganisms, which includes *Klebsiella pneumoniae*, is characterized by a high frequency of resistance to various antibiotics [6]. Therefore, in this work, bacteriophages were isolated specifically for this bacterium.

3 out of 4 studied bacteriophages formed lysis spots with halos. Halo formation is often associated with the action of polysaccharide depolymerases (endo-glycanohydrolases) [65]. It is known that the presence of polysaccharide-degrading enzymes is a favorable factor for phage therapy, as they contribute to the destruction of capsules and biofilms [66]. Their action facilitates access to bacterial cells of both bacteriophages and cells of the immune system, as well as drugs [32]. The annotation results of bacteriophage genomes confirmed the relationship of the action of polysaccharide depolymerases with the formation of halo, which was also described in many works [61, 62, 67, 68]. Bacteriophage vB_KpnS_FZ41, which doesn’t encode any polysaccharide depolymerases, had significantly different host range from the bacteriophages vB_KpnS_FZ10, vB_KpnP_FZ12 and vB_KpnM_FZ14 that encode polysaccharide depolymerases. Together, a combination of all 4 bacteriophages lysed all the studied strains of *Klebsiella pneumoniae*. This data indicates that both phages with and without the presence of polysaccharide-degrading enzymes should be included in bacteriophage cocktails, because it can broaden their lytic spectra and increase the efficiency of therapeutic phage cocktails.

Previously obtained data on the study of the lytic spectra of 32 *Klebsiella pneumoniae* bacteriophages [69] showed that phages of *Siphoviridae* and *Podoviridae* families lyse from 7 to 15% of strains, only one phage (*Podoviridae*) was effective against 22% of strains. Bacteriophages of *Myoviridae* family were active against 4–22% of *Klebsiella pneumoniae* strains. Bacteriophages vB_KpnS_FZ10, vB_KpnS_FZ41, vB_KpnP_FZ12 and vB_KpnM_FZ14 showed rather high lytic activity, which confirms their potential for prophylaxis and treatment of bacterial infections.

However, we focus not on the individual lytic properties of bacteriophages, but on their effect in the “cocktail”. Studying the frequency of generation of phage-resistant forms has shown that it ranges from $4 \times 10^{ - 7}$ to $9 \times 10^{ - 7}$, depending on the virus. The best result was obtained for a combination of bacteriophages vB_KpnS_FZ10, vB_KpnP_FZ12 and vB_KpnM_FZ14—not a single resistant colony was found. Our bacterial collection does not contain a strain that is sensitive to all 4 phages at once, so the cocktail for establishing the frequency of phage-resistant forms generation was composed of 3 bacteriophages. Thus, the use of phage cocktails in therapy allows not only to increase the lytic effectiveness of the preparation, but also significantly reduce the risk of phage-resistant forms formation.

Prophages were not inducible from phage-resistant colonies under experimental conditions. To exclude possible assumptions about the temperate nature of studied bacteriophages, their genomes were sequenced. This is a prerequisite for the inclusion of phages in the preparation, since the presence of moderate viruses is undesirable for therapy [40]. Sequencing data confirmed the lytic nature of all investigated bacteriophages, since the genes responsible for lysogeny are not encoded in their genome.

Based on the differences in morphology and lytic spectra, we can assume that all phages have an affinity for different surface structures on the host bacterium. The tail fiber proteins of all viruses studied in this work differ in their folding and domain structure, which was established during the analysis of homologs of structural proteins from the UniProt base (vB_KpnS_FZ10—A0A4D6T3L6; vB_KpnP_FZ12—A0A4D6T3P7; vB_KpnM_FZ14—A0A4D8SZG4; vB_KpnS_FZ41—A0A4D6T3Y8).

Biophysical stability characteristics correlate with the known published data on *Klebsiella pneumoniae* phages belonging to the families *Siphoviridae*, *Myoviridae* and *Podoviridae* [41, 69], as well as with earlier studies by Ackermann and Dubow [70], which suggested that most phages are able to maintain their activity in wide pH ranges (5–9) under physiological conditions, and inactivation at pH 1–3 can be associated with denaturation of virion proteins under acidic conditions [71]. Moreover, this range of resistance indicates high stability of all bacteriophages, which suggests the possibility of titer maintenance under storage conditions and in therapeutic use both in the urogenital area and for treatment of pulmonary infections.

Recommendations for the selection of therapeutic phages state that 70% of particles or more should be adsorbed in the first 10 min, and the adsorption constant should be 10^–8^—10^–9^ ml/min [72]. Adsorption constants of studied phages (10^–9^) confirm their perspective for therapy and prevention of bacterial infections.

Latent period of studied phages correlates with other data on bacteriophages of the family *Siphoviridae* [41] and *Myoviridae* [69]. Latent period of vB_KpnP_FZ12 was 2 times longer than that of *Podoviridae* representatives in Kęsik-Szeloch et al. [69], however, the phage burst size in the case of vB_KpnP_FZ12 is 1.5 times higher. Burst size was lower than that of representatives of the *Siphoviridae* family in Jamal et al. [41] study, but higher than in the data of Kęsik-Szeloch et al. [69] for the families *Siphoviridae*, *Myoviridae*, and *Podoviridae*.

The obtained titers during the growth of phages in a liquid nutrient medium suggest that such values ​​of the adsorption constant, latent period, and phage burst size provide effective inhibition of *Klebsiella pneumoniae* culture growth, and indicate sufficient virus productivity for obtaining high concentrations in the final preparation.

Restriction analysis of viral DNA showed that the bacteriophage vB_KpnS_FZ10 adapted to restriction-modification systems of host bacteria. In addition to the lack of sites for the restriction enzyme of *Klebsiella pneumoniae* (KpnI), as well as for HindIII and SmaI, vB_KpnS_FZ10 has additional protection systems and encodes its own adenine methyltransferase and cytosine methyltransferase. Bacteriophage KP36 from *Siphoviridae* family [69] has similar properties. Sequencing data showed that phages vB_KpnS_FZ10 (MK521904) and KP36 (JF501022) have a high level of homology and probably belong to the same genus *Webervirus*.

Bacteriophage vB_KpnP_FZ12 is also an example of adaptation to host restriction-modification systems by the loss of restriction sites, its DNA does not contain recognition sites for HindIII, SspI, BamHI and EcoRI restriction enzymes. The closely related bacteriophage KP32 [69] had similar properties. Sequencing data showed that the phages vB_KpnP_FZ12 (MK521905) and KP32 (GQ413937) have a high level of homology and probably belong to the same genus *Pzondovirus*. The bacteriophages vB_KpnS_FZ41 and vB_KpnM_FZ14 were found to be sensitive to almost all bacterial enzymes used, including the restriction enzyme KpnI. For NotI restriction enzyme in silico, the presence of cutting sites in the vB_KpnS_FZ10 genome was predicted, for MspI in the vB_KpnS_FZ41 genome, and for NdeI in the vB_KpnM_FZ14 genome, however, restriction analysis did not show the effect of these enzymes. The differences between the obtained restriction profiles and those predicted in silico cannot be explained by the insufficient quality of the enzymes used, since they acted on the genomes of the remaining phages. Restriction enzymes are also resistant to Dam and Dcm methylation, so the absence of cutting cannot be associated with the action of adenine/cytosine methyltransferases, however, phage genomes are characterized by a number of modifications besides Dam and Dcm methylation [73], therefore, deviation from the predicted restriction profiles can be due to base modification.

During annotation, it was found that the bacteriophage vB_KpnS_FZ41 encodes its own tRNAs. It has been shown that some phages with a sufficiently large coding capacity can use their own tRNAs for codons, which are much more common in their genome than in the host bacterium [74].

## Conclusions

The obtained host range, biophysical stability, burst size, latent period and genome data, together with the presence of depolymerases, indicate that the studied bacteriophages are promising for further in vivo studies and might be used in phage therapy as part of a complex therapeutic and prophylactic phage preparation. The conducted studies showed that the complex preparation is more effective than individual phages. The use of the complex phage cocktail allows to extend the lytic spectrum, and significantly reduces the possibility of phage-resistant forms generation.

## Supplementary Information

**Additional file 1.** Klebsiella pneumoniae strains classification.Legend. Classification was carried out with MALDI-TOF Microflex mass spectrometer and biochemical tests (MIKROLATEST) with further analysis on Multiskan Ascent spectrophotometer. Top hit from MALDI Biotyper Classification Results is presented.

**Additional file 2.** Detailed genome maps of bacteriophages vB_KpnS_FZ10, vB_KpnS_FZ41, vB_KpnP_FZ12 and vB_KpnM_FZ14. Legend. Genome maps were visualized with GenomeVx [54] based on complete genome sequences of Klebsiella pneumoniae phages vB_KpnS_FZ10, vB_KpnP_FZ12, vB_KpnM_FZ14 and vB_KpnS_FZ41, deposited in GenBank under the accession numbers MK521904, MK521905, MK521906 and MK521907, respectively.

## Data Availability

The complete genome sequences of *Klebsiella pneumoniae* phages vB_KpnS_FZ10, vB_KpnP_FZ12, vB_KpnM_FZ14 and vB_KpnS_FZ41 have been deposited in GenBank under the accession numbers MK521904, MK521905, MK521906 and MK521907, respectively. Raw Illumina reads are available on NCBI SRA under accession numbers SRR10037530, SRR10037529, SRR10037528 and SRR10037527, respectively. The associated BioProject accession number is RJNA562287.

## Abbreviations

ESKAPE microorganisms: *Enterococcus faecium*, *Staphylococcus aureus*, *Klebsiella pneumoniae*, *Acinetobacter baumannii*, *Pseudomonas aeruginosa* And *Enterobacter*
ESBL: Extended-Spectrum Beta Lactamase
BHI: Brain Heart Infusion
CFU: Colony Forming Units
PFU: Plaque Forming Units
MPB: Meat-Peptone Broth
EDTA: Ethylenediaminetetraacetic Acid
SDS: Sodium Dodecyl Sulfate
ORF: Open Reading Frame
