# Supplementary figures and images for: Characterization of four virulent Klebsiella pneumoniae bacteriophages, and evaluation of their potential use in complex phage preparation
Source: Virol J. 2021 Jan 6;18:9. doi: 10.1186/s12985-020-01485-w (PMC7789013; doi:10.1186/s12985-020-01485-w)

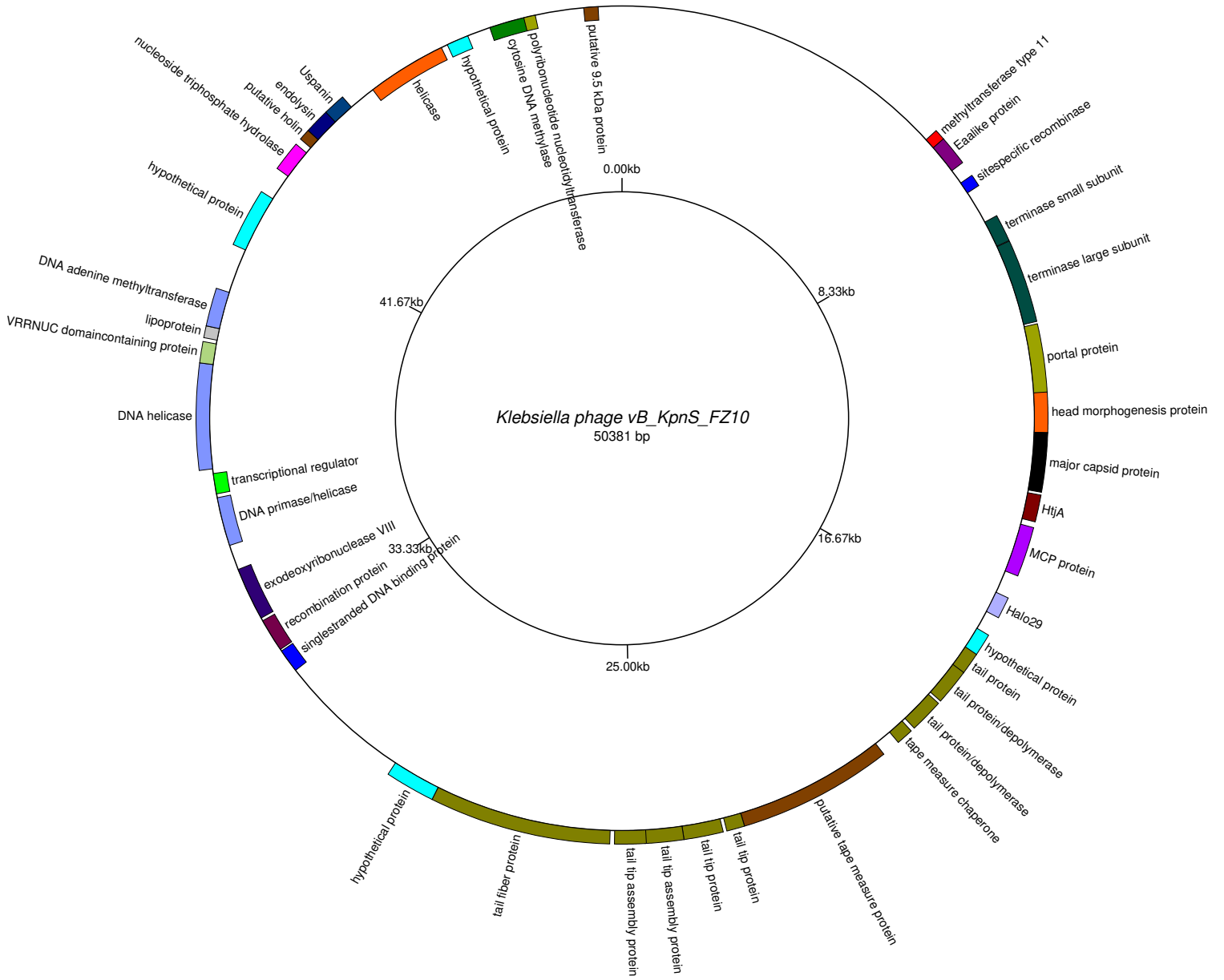

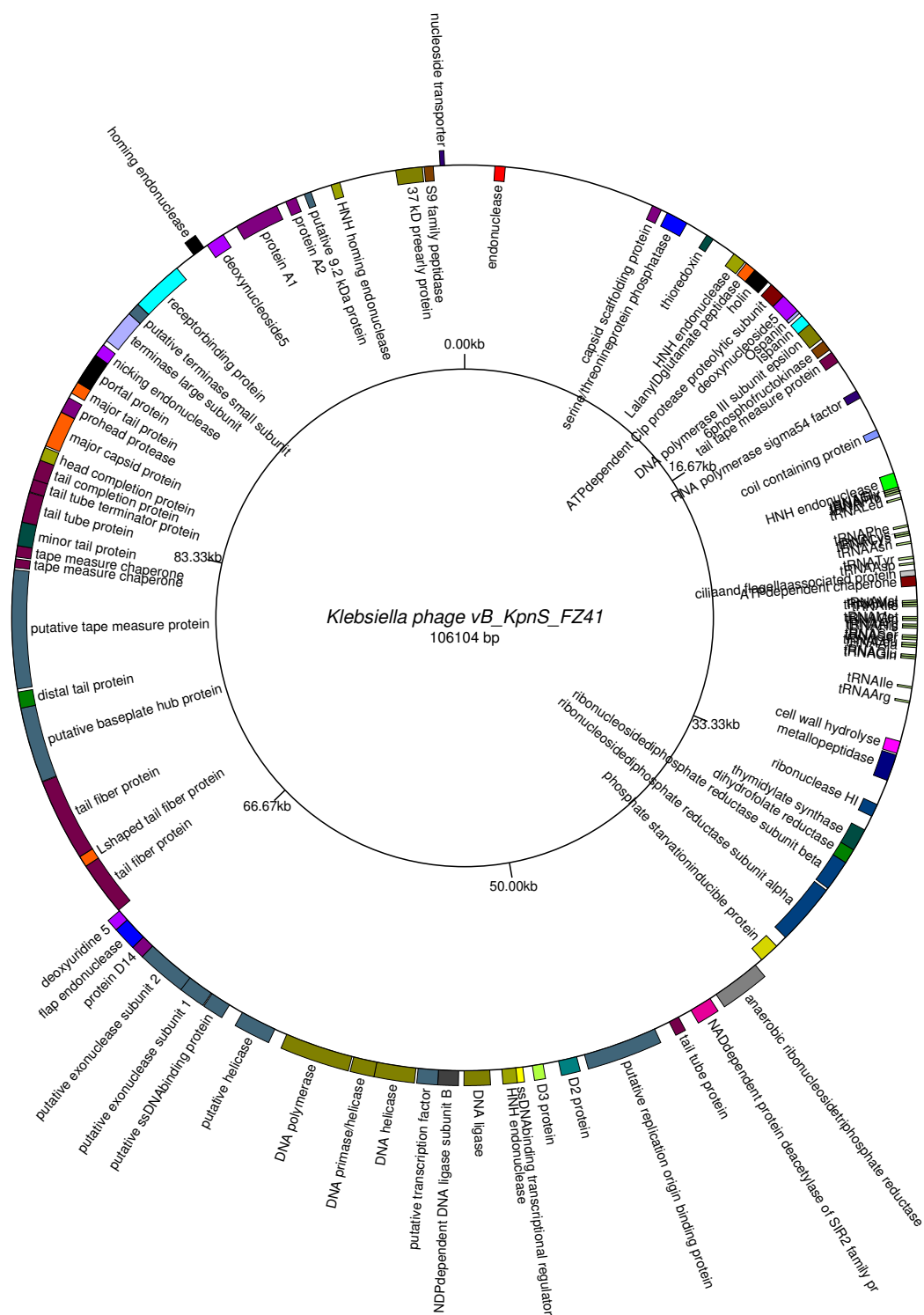

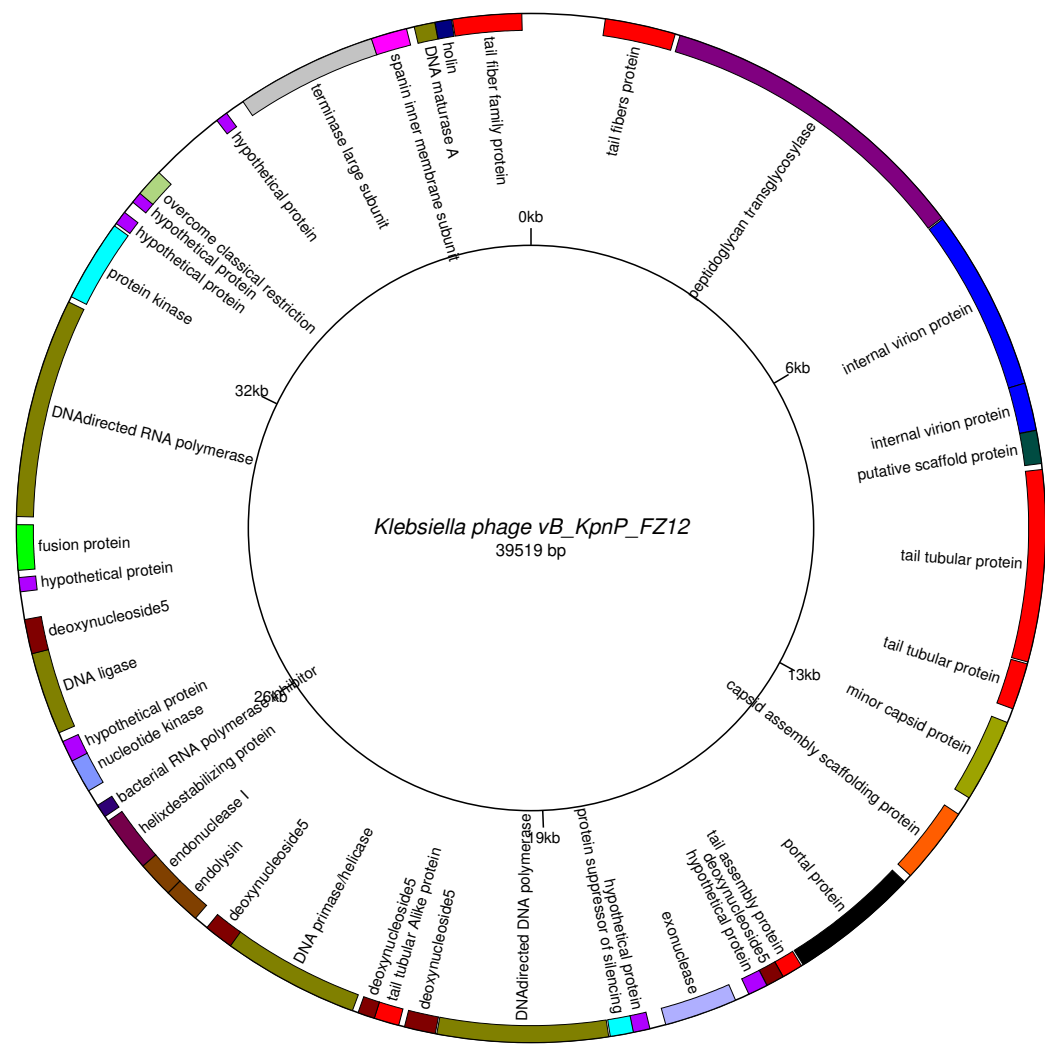

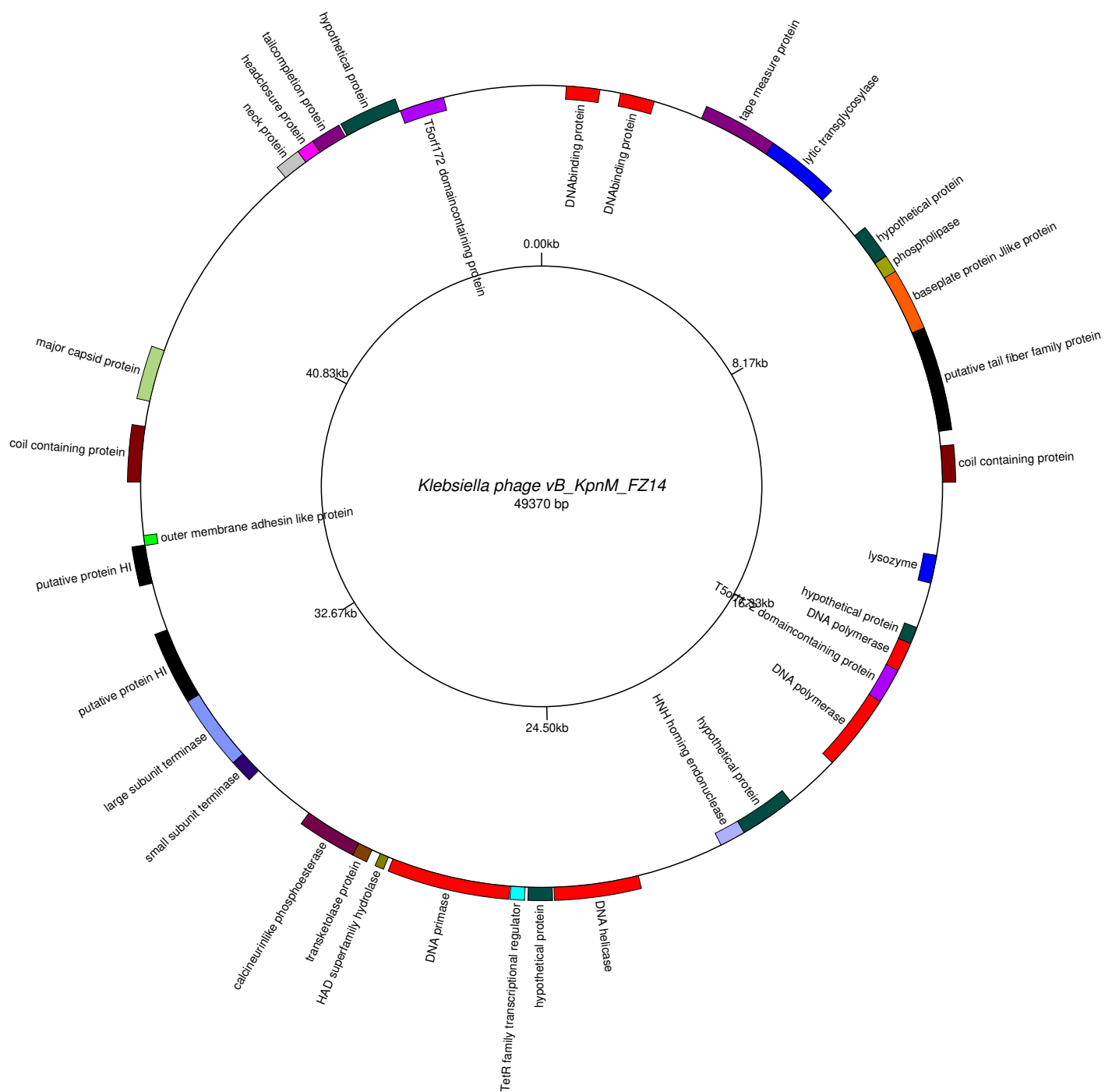

Supplement: Supplementary file 2 — Additional file 2. Detailed genome maps of bacteriophages vB_KpnS_FZ10, vB_KpnS_FZ41, vB_KpnP_FZ12 and vB_KpnM_FZ14. Legend. Genome maps were visualized with GenomeVx [54] based on complete genome sequences of Klebsiella pneumoniae phages vB_KpnS_FZ10, vB_KpnP_FZ12, vB_KpnM_FZ14 and vB_KpnS_FZ41, deposited in GenBank under the accession numbers MK521904, MK521905, MK521906 and MK521907, respectively. [file 12985_2020_1485_MOESM2_ESM.pdf]
